# 1702. Platelet Counts in Contezolid Complicated Skin and Soft Tissue Infection Phase 2 and Phase 3 Clinical Trials

**DOI:** 10.1093/ofid/ofac492.1332

**Published:** 2022-12-15

**Authors:** Edward Fang, Huahui Yang, Hong Yuan

**Affiliations:** MicuRx Pharmaceuticals Inc, San Carlos, California; MicuRx Pharmaceuticals Inc, San Carlos, California; MicuRx Pharmaceuticals Inc, Foster City, CA, USA, shanghai, Shanghai, China

## Abstract

**Background:**

Contezolid (CZD; MRX-I) is a novel oral (PO) oxazolidinone with potent activity against Gram-positive pathogens, including methicillin-resistant *Staphylococcus aureus* (MRSA) and vancomycin-resistant *Enterococccus* (VRE). Nonclinical and Phase 1 (Ph1) clinical data indicate CZD may cause less myelosuppression, particularly with longer duration therapy, and with reduced risk of monoamine oxidase inhibition compared with linezolid (LZD). In Phase 2 (Ph2) and Phase 3 (Ph3) complicated skin and soft tissue infections (cSSTI) clinical trials, CZD was compared with LZD with dosing for 7-14 days, and noninferiority was demonstrated in the Ph3 study. Overall safety was comparable to LZD, and the most common treatment emergent adverse events (TEAEs) in both the CZD and LZD groups were gastrointestinal; however, hematologic laboratory abnormalities and TEAEs were less common with CZD. In June 2021, CZD was approved in China for cSSTI. Sequential therapy with intravenous contezolid acefosamil (CZA; double prodrug of CZD) followed by CZD PO is being evaluated in global Ph3 diabetic foot infection (DFI) and acute bacterial skin and skin structure infection (ABSSSI) clinical trials. Because DFI requires longer treatment (14-28 days), changes in platelet counts with therapy ≥11 days were evaluated in completed Ph2 and Ph3 CZD cSSTI studies.

**Methods:**

In Ph2 and Ph3 cSSTI studies, mean percent changes in platelet counts from baseline (BSL) to the end of therapy (EOT) visit were compared for all subjects in the safety analysis sets (SS) who received CZD 800 mg and LZD, and also the subjects who received ≥11 days of therapy.

**Figure:**
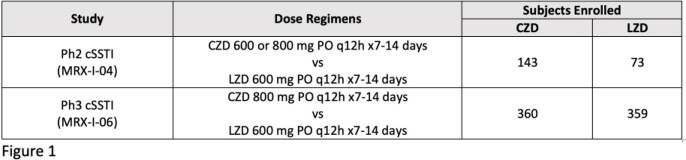

**Results:**

In the Ph2 and Ph3 cSSTI studies, the mean platelet count values for LZD subjects decreased compared with CZD subjects, and the difference was greater in subjects who received ≥11 days of therapy.

**Figure:**
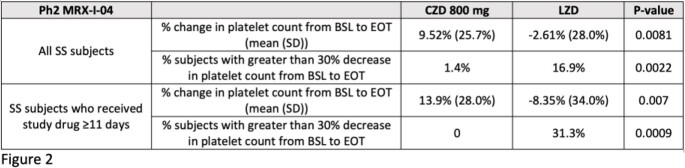

**Figure:**
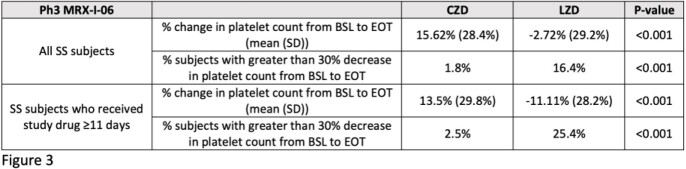

**Conclusion:**

In Ph2 and Ph3 cSSTI clinical trials with treatment durations of 7-14 days, mean platelet counts did not decrease for CZD subjects while mean values for LZD subjects did decline, consistent with nonclinical and Ph1 clinical data. Differences were more significant in subjects who received ≥11 days of therapy. In the current Ph3 global DFI study which compares 14-28 days of CZA/CZD to LZD, evaluation of hematological safety is an important outcome measure.

**Disclosures:**

**Edward Fang, MD**, MicuRx Pharmaceuticals Inc: Employee **Huahui Yang, MS**, MicuRx Pharmaceuticals Inc: Employee.

